# Artificial intelligence for assessing the severity of microtia *via* deep convolutional neural networks

**DOI:** 10.3389/fsurg.2022.929110

**Published:** 2022-09-08

**Authors:** Dawei Wang, Xue Chen, Yiping Wu, Hongbo Tang, Pei Deng

**Affiliations:** Department of Plastic and Cosmetic Surgery, Tongji Hospital of Tongji Medical College of Huazhong University of Science and Technology, Wuhan, China

**Keywords:** artificial intelligence, microtia, severity, convolutional neural networks, objective

## Abstract

**Background:**

Microtia is a congenital abnormality varying from slightly structural abnormalities to the complete absence of the external ear. However, there is no gold standard for assessing the severity of microtia.

**Objectives:**

The purpose of this study was to develop and test models of artificial intelligence to assess the severity of microtia using clinical photographs.

**Methods:**

A total of 800 ear images were included, and randomly divided into training, validation, and test set. Nine convolutional neural networks (CNNs) were trained for classifying the severity of microtia. The evaluation metrics, including accuracy, precision, recall, F1 score, receiver operating characteristic curve, and area under the curve (AUC) values, were used to evaluate the performance of the models.

**Results:**

Eight CNNs were tested with accuracy greater than 0.8. Among them, Alexnet and Mobilenet achieved the highest accuracy of 0.9. Except for Mnasnet, all CNNs achieved high AUC values higher than 0.9 for each grade of microtia. In most CNNs, the grade I microtia had the lowest AUC values and the normal ear had the highest AUC values.

**Conclusion:**

CNN can classify the severity of microtia with high accuracy. Artificial intelligence is expected to provide an objective, automated assessment of the severity of microtia.

## Introduction

Microtia is a congenital abnormality with an estimated incidence of 0.83–17.4 per 10,000 births (1). Microtia generally presents as auricle malformation, varying from slight structural abnormalities to complete absence of the external ear (2). Moreover, congenital aural atresia or stenosis usually occurs together with microtia. Surgical auricular reconstruction using autologous costal cartilage has been widely performed since it was first reported by Tanzer (3). Then, the two-stage reconstruction procedure was developed by Brent (4) and Nagata (5). Nevertheless, auricular reconstruction remains one of the most complex and challenging procedures for plastic surgeons.

The severity of microtia can influence the surgical procedures and postoperative outcome of auricular reconstruction. To date, various classifications have been introduced for assessing the severity of microtia by Marx, Lapchenko, Gill, Rogers, Tanzer, Jahrsdoerfer, and Tasse et al. (6). However, these classification methods all rely on subjective assessment of auricular malformation, and no reliable objective method has been proposed to assess the severity of microtia. An objective diagnostic tool will provide a standardized, repeatable assessment of the severity of microtia, avoiding inconsistency between providers.

Artificial intelligence has been increasingly applied in various fields of medicine, particularly in the detection of skin diseases (7), breast cancer (8), oral cancer (9), and diabetic retinopathy (10). Convolutional neural networks (CNNs), as the most essential algorithms for deep learning, facilitate the development of artificial intelligence in image recognition, classification, and detection. In the field of plastic surgery, several studies have explored the effectiveness of CNN models in assessing the severity of unilateral cleft lip (11) and evaluating age reduction after face-lift (12). Due to the complex shape and composition of the ear, it is difficult to develop mathematical models to identify deformities in the ear. Previous studies have developed CNN models to identify ear abnormalities from two-dimensional photographs with satisfactory results (13–15). To our knowledge, no CNN model has been reported to evaluate the severity of microtia, for the reported models only distinguished between normal ears and malformed ears.

**Figure 1:**
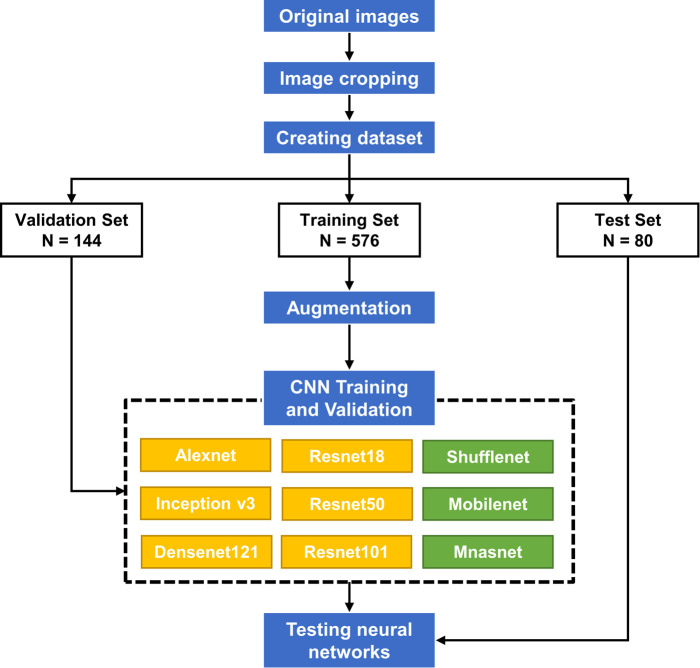
The schematic diagram of the whole process.

Thus, we hypothesized that CNN models have the potential to accurately classify the severity of microtia using two-dimensional photographs. In this study, we trained nine CNN models and evaluated their performance in assessing the severity of microtia using clinical photographs. The design of this study was illustrated in Figure 1. This study aimed to recommend better CNNs from the tested networks for clinical application, which would facilitate the objective classification of microtia.

## Methods

### Image datasets

The image datasets were collected retrospectively from our hospital between January 2015 and June 2021 in this study. A total of 800 ear images (left or right side) were included, consisting of 360 images of normal ear and 440 images of microtia. Lateral view photographs of the ears were captured using a digital camera (Nikon D3100, Tokyo, Japan). The inclusion criteria were microtia or normal ears without previous ear surgery. The images that seem blurred or at undesired angulation were excluded. This study was approved by the local Ethical Committee (TJ-IRB20220112). The committee waived the need for individual informed consent as the study was retrospective and non-interventionist.

### Severity assessment of microtia

**Figure 2:**
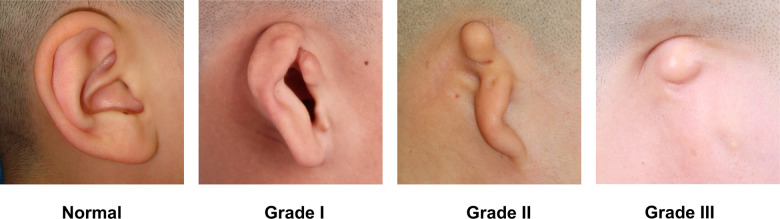
The severity assessment of microtia.

The severity of microtia was classified according to the classification used by Mastroiacovo (16). Grade I microtia corresponded to concha-type microtia with a small auricle and some distinguishable anatomic structures (Figure 2). Grade II microtia as lobule-type microtia showed a residual vertical ridge of tissue shaped like a peanut. Grade III microtia was almost or complete absence of the pinna. The grades of 800 ear images were labeled by two experienced plastic surgeons.

### Pre-processing

The images were cropped to the ear boundary at a size of 1:1 and then used for deep learning. A total of 800 images were randomly divided into training, validation, and test sets with 640, 80, and 80 images, respectively (Figure 1). The training and validation sets were used to train CNN models, and the test set provided the final evaluation of the model's performance. To reduce overfitting due to the small dataset size, online data augmentation was applied to increase the data size. The training images were randomly flipped, rotated, and displaced to achieve data augmentation.

### CNN models and training

Nine CNNs, including Alexnet, Inception v3, Densenet121, Resnet18, Resnet50, Resnet101, Shufflenet v2, Mobilenet v2, and Mnasnet were used for classifying the severity of microtia. The basic properties of the CNNs are listed in Table 1. Alexnet, Resnet101, Resnet50, Inception v3, Resnet18, and Densenet121 are relatively large networks, of which Alexnet is the largest with over sixty million parameters. Shufflenet, Mobilenet, and Mnasnet are relatively small networks with less than four million parameters. The training process consisted of forward propagation and backward propagation. With images input into the networks, forward propagation output the classification result, including the accuracy and loss between the predicted and true labels. Then, the model parameters were updated through backward propagation. The models were trained up to 100 epochs with 16 mini-batch sizes and established based on the maximum accuracy and minimum loss in the validation set.

**Table 1:** The properties of the convolutional neural networks.

Networks	Depth	Size (MB)	Parameters (millions)	Image input size
Alexnet	8	227	61.1	256*256*3
Inception v3	48	89	23.9	299*299*3
Densenet121	121	56	8.1	224*224*3
Resnet18	18	44	11.7	224*224*3
Resnet50	50	96	25.6	224*224*3
Resnet101	101	167	44.6	224*224*3
Shufflenet	50	5	1.4	224*224*3
Mobilenet	54	13	3.5	224*224*3
Mnasnet	18	14	3.9	224*224*3

### Performance evaluation

The evaluation metrics, including accuracy, precision, recall, F1 score were calculated with the confusion matrix of the test set. Additionally, the receiver operating characteristic (ROC) curve and area under the ROC curve (AUC) were used to assess the diagnostic performance of the CNN models. The calculation formulas for the evaluation metrics were as follows (TP: true positive, TN: true negative, FP: false positive, FN: false negative):

Accuracy=(TP+TN)/(TP+FP+FN+TN)

Precision=TP/(TP+FP)

Recall=TP/(TP+FN)

F1score=2∗(Precision∗Recall)/(Precision+Recall)

## Results

Of the total 800 ear images, 360, 150, 240, and 50 images were classified as normal ears, grade I microtia, grade II microtia, and grade III microtia, respectively. The test set was composed of 30 images of normal ears, 20 images of grade I microtia, 20 images of grade II microtia, and 10 images of grade III microtia.

**Figure 3:**
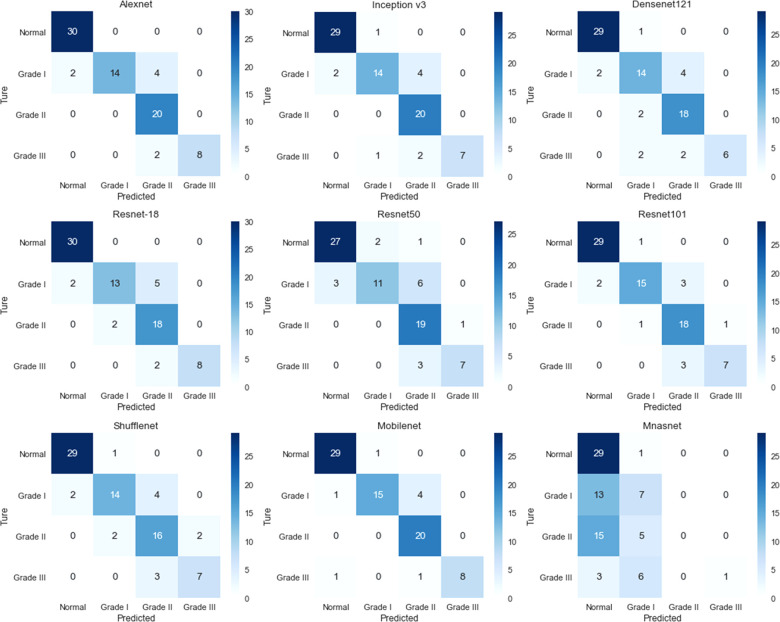
The confusion matrix of the convolutional neural networks for assessing the severity of microtia.

The training loss and validation accuracy of the nine CNNs were shown in Supplementary Figures S1, S2, respectively. The confusion matrices for each network between true and predicted labels of the test set were presented in Figure 3. The evaluation metrics of each network in the test set, including accuracy, precision, recall, and F1 score, were summarized in Table 2. Eight CNNs were tested with accuracy more than 0.8, while Mnasnet only reached 0.463. Among them, Alexnet achieved a relatively high accuracy of 0.900, a precision of 0.927, a recall of 0.875, and an F1 score of 0.897. As a small network, Mobilenet also achieved an accuracy of 0.900, a precision of 0.918, a recall of 0.879, and an F1 score of 0.898.

**Table 2:** The performance of convolutional neural networks for assessing the severity of microtia.

Networks	Accuracy	Precision	Recall	F1-Score
Alexnet	0.900	0.927	0.875	0.897
Inception v3	0.875	0.895	0.842	0.871
Densenet121	0.838	0.856	0.792	0.834
Resnet18	0.863	0.881	0.837	0.860
Resnet50	0.800	0.819	0.775	0.795
Resnet101	0.863	0.861	0.829	0.861
Shufflenet	0.825	0.808	0.792	0.824
Mobilenet	0.900	0.918	0.879	0.898
Mnasnet	0.463	0.463	0.354	0.354

**Figure 4:**
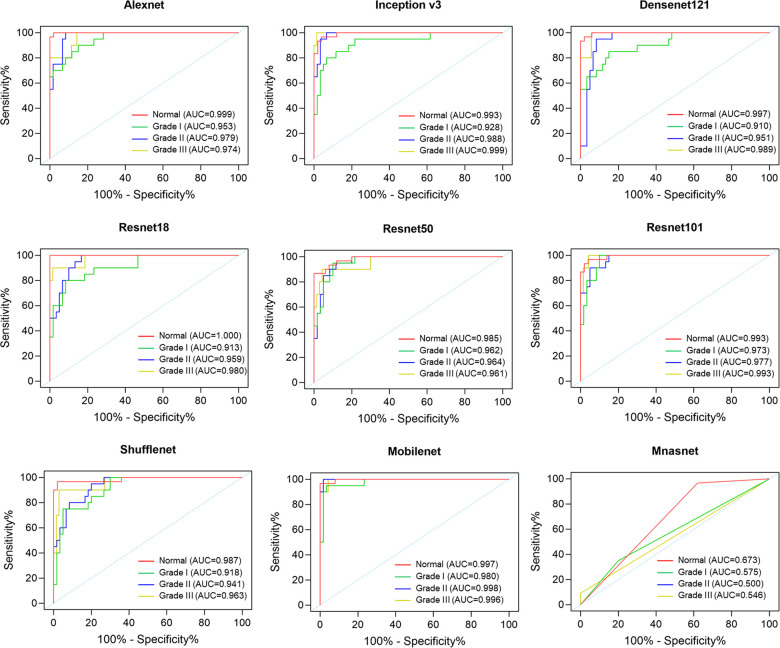
The ROC curves of the convolutional neural networks for assessing the severity of microtia.

ROC curves and AUC values were obtained for each grade of microtia corresponding to each CNN (Figure 4). All CNNs except Mnasnet achieved high AUC values more than 0.9 for each grade of microtia. Particularly in the Alexnet, Resnet50, Resnet101, and Mobilenet, the AUC values for each grade of microtia were greater than 0.95. In most CNNs, such as Alexnet, Inception v3, Densenet121, Resnet18, Resnet101, and Shufflenet, the grade I microtia had the lowest AUC values and the normal ear had the highest AUC values.

## Discussion

In recent years, artificial intelligence has attracted increasing attention in healthcare. As an important technique of artificial intelligence, machine learning is used for data analysis that enables computers to learn from data, extract meaningful patterns and make reliable decisions (17). Deep learning, a subset of machine learning, can extract higher-level features from data using neural networks. Among deep learning methods, convolutional neural networks (CNNs) have made considerable progress in image analysis, such as image recognition, classification, and detection, attributed to their deep layer structure (18).

The appearance of the outer ear is characterized by morphological features, including tragus, lobule, antitragus, concha, helix, antihelix, scapha, navicular fossa, and other structures. Besides face, iris and fingerprints, ear allows for personal identification due to a large number of unique features. Recently, several studies have demonstrated the effectiveness of CNNs in ear recognition with high recognition rates (19–22). Additionally, studies have reported that CNNs can identify normal ear and congenital ear abnormalities from two-dimensional photographs (13–15). Hallac et al. found that the CNN GoogLeNet could serve as an ear deformity detection model with a test accuracy of about 94.1% (13). Besides, CNN was demonstrated to be a robust tool for assessing outcomes of ear molding therapy by removing the subjectivity of human evaluation (14). Similarly, Ye et al. revealed that the CNN ResNet can evaluate reconstructed auricles in a manner resembling that of a medical student, indicating the potential of CNN for assessing the outcomes of auricular reconstruction (15). Overall, CNN has the ability to identify the morphological structure of the ear.

Microtia is a common congenital disease in plastic surgery, however, no reliable objective method exists to assess its severity. There is no gold standard for the severity of microtia, although multiple classifications have been introduced relying on subjective assessment. The complexity of the ear makes it difficult to establish mathematical models to assess microtia. To our knowledge, there is no previous study to assess the severity of microtia using CNN.

In this study, we tested nine CNNs that have been applied and performed well in medical image classification (23–28). The Alexnet architecture won the 2012 ImageNet Large Scale Visual Recognition Challenge, and since then CNNs have been flourishing. The innovations of the Inception-v3 architecture were factorized convolutions and aggressive regularization to improve classification accuracy. Then, the Resnet architecture was proposed to learn residual functions with reference to the layer inputs instead of learning unreferenced functions. Subsequently, Densenet extended the network connectivity on the basis of Resnet by connecting each layer to other layers in a feed-forward fashion. Additionally, Shufflenet, Mobilenet, and Mnasnet were lightweight neural networks and specialized toward use in mobile devices.

The dataset in this study was relatively small, which could lead to the overfitting of the CNN model. Therefore, the technique of data augmentation was performed to increase the performance of the CNN models. We found that most CNN models achieved high accuracy over 0.8 in assessing the severity of microtia, particularly Alexnet and Mobilenet even achieved a high accuracy of 0.9. In general, the deeper the CNN, the higher the accuracy. However, this trend was not obvious in this study, where the depth of network was not proportional to the classification accuracy. The results showed that Resnet50 and Resnet101 were no more accurate than Resnet18, indicating adequate learning could still be achieved despite the small number of layers. Similarly, this study did not reveal a positive correlation between the number of parameters in CNN and classification accuracy. Although Shufflenet and Mobilenet were lightweight networks with less than 4 million parameters, the accuracy remained high in this study. Regarding the accuracy of each grade of microtia in the CNNs, the AUC values for grade I microtia were relatively low compared to the other grades. It is presumed that grade I microtia varies widely from near normal to near peanut-shaped auricles, resulting in lower classification accuracy.

An ideal diagnostic tool for microtia needs to be objective, reproducible, low-cost, simple to implement, and allow for real-time feedback. The findings in the present study demonstrate the high accuracy of CNNs in classifying microtia, even with a lightweight CNN. Therefore, to make the use convenient and practical in clinical situations, the CNN models are available for mobile platforms, such as smartphones, with relatively small memory size and computing power. In clinical use, a doctor or non-professional can take a photo of the ear using mobile devices, and the machine will automatically provide real-time feedback on the severity of the microtia. Importantly, the performance of the CNN model can continue to improve, with the development of algorithms and the expansion of training data.

Some limitations exist in this study. Firstly, a relatively small data set of 800 clinical images was included, and a large number and high-quality images from multicenter are required for better-performing CNN models. Secondly, due to the absence of a gold standard for assessing the severity of microtia, we have adopted a popular classification as ground truth based on subjective observation of the auricular shape. Although this classification method may not be accepted by all surgeons, our results indicate deep learning can be a feasible and automated method for microtia severity. Thirdly, current classification methods mostly rely on subjective assessment by surgeons, and no reliable objective method to evaluate the severity of microtia has been reported before. The lack of comparison with state of art methods is another limitation of our study. Finally, images were cropped manually in the pre-processing, which required automation of image acquisition and pre-processing when developing diagnostic tools for clinical use.

## Conclusion

Artificial intelligence is a potentially practical method to objectively assess the severity of microtia. This study tested the performance of nine CNNs for classifying microtia, and most CNN models possessed high accuracy, even with lightweight networks and insufficient training images. The CNN models applied to mobile platforms could be a more available and standardized tool in future clinical practice.

## Data Availability

The original contributions presented in the study are included in the article/**Supplementary Material**, further inquiries can be directed to the corresponding author/s.
