# Supplementary figures and images for: Artificial intelligence for assessing the severity of microtia via deep convolutional neural networks
Source: Front Surg. 2022 Sep 8;9:929110. doi: 10.3389/fsurg.2022.929110 (PMC9492961; doi:10.3389/fsurg.2022.929110)

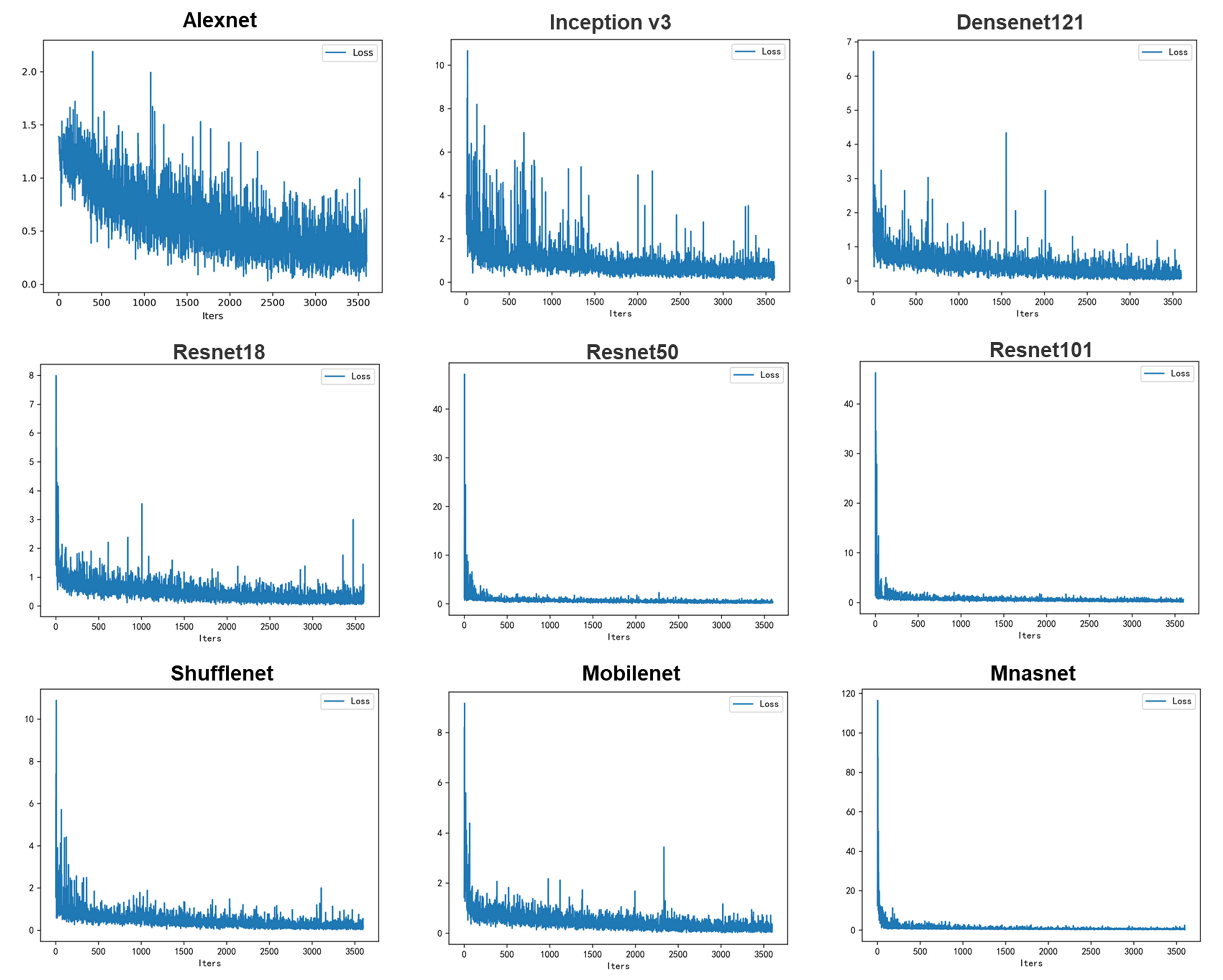

Supplement: Supplementary file 1 [file Supplementary_Figure_1.tif]

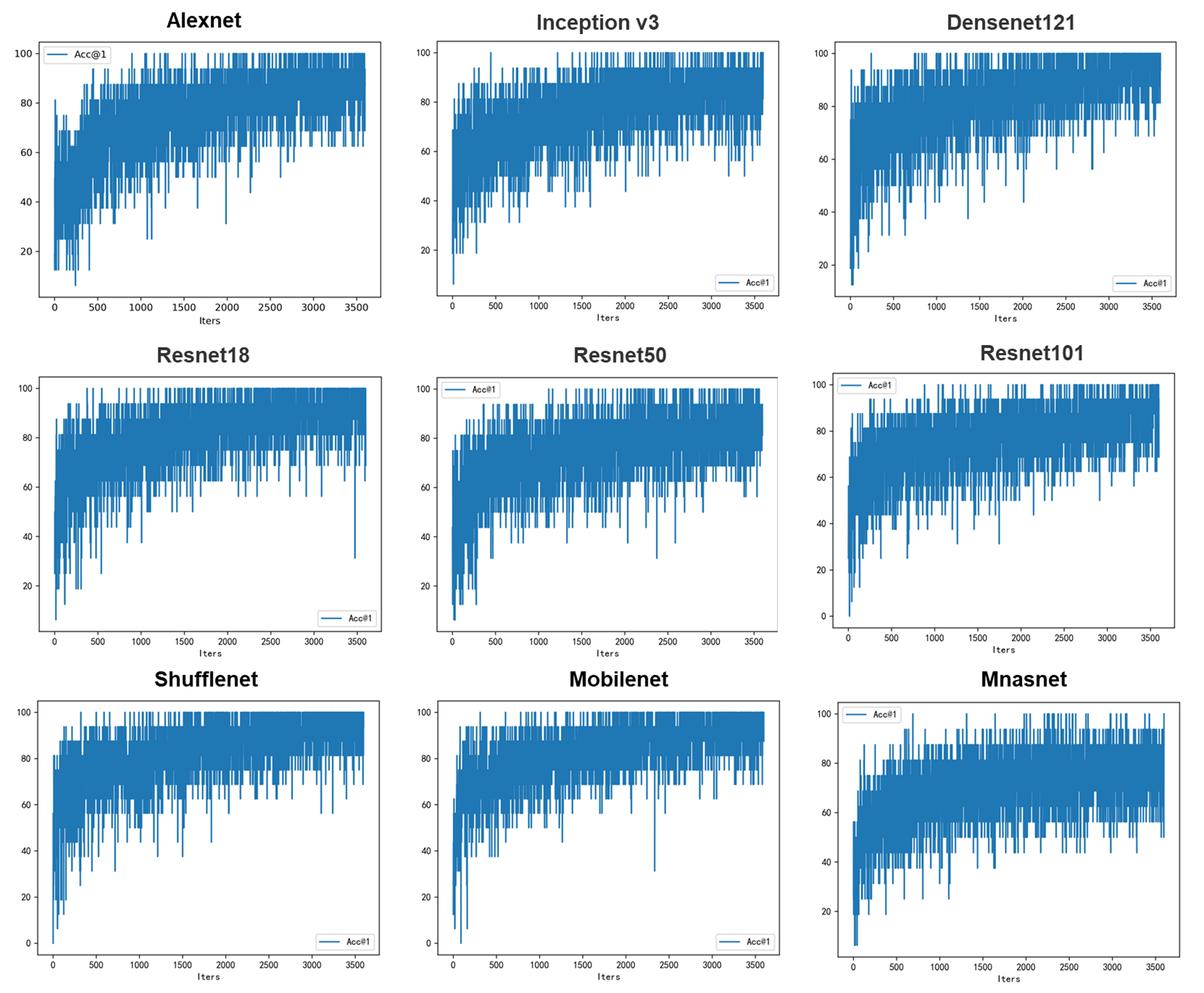

Supplement: Supplementary file 2 [file Supplementary_Figure_2.tif]
