# Supplementary material for: Artificial intelligence for assessing the severity of microtia via deep convolutional neural networks
Source: Front Surg. 2022 Sep 8;9:929110. doi: 10.3389/fsurg.2022.929110 (PMC9492961; doi:10.3389/fsurg.2022.929110)
Supplement: Supplementary file 3 [file Appendix.docx]

**Appendix**

**Supplementary Figure 1** The training loss of the nine convolutional neural networks

**Supplementary Figure 2** The validation accuracy of the nine convolutional neural networks
